# *Toxoplasma gondii* and *Neospora caninum* antibody seroprevalence and risk factors among dogs treated at Public Veterinary Hospitals in São Paulo, Brazil

**DOI:** 10.1590/S1984-29612023058

**Published:** 2023-11-10

**Authors:** Elidia Zotelli dos Santos, Herbert Souza Soares, Stephanie Rodrigues dos Santos, Jonas Moraes, Hilda Fátima de Jesus Pena, Marcos Amaku, Solange Maria Gennari

**Affiliations:** 1 Programa de Pós-graduação em Saúde Única, Faculdade de Medicina Veterinária, Universidade Santo Amaro – UNISA, São Paulo, SP, Brasil; 2 Faculdade de Medicina Veterinária e Zootecnia, Universidade de São Paulo – USP, São Paulo, SP, Brasil; 3 Faculdade de Medicina, Universidade de São Paulo – USP, São Paulo, SP, Brasil

**Keywords:** Protozoa, dogs, risk factors, coccidia, Protozoários, cães, fatores de risco, coccídios

## Abstract

Dogs can be infected by *Toxoplasma gondii* and *Neospora caninum*, for which they function, respectively, as intermediate, and definitive hosts. In the present study seroprevalence against *T. gondii* and *N. caninum* antibodies, were determined by indirect fluorescent antibody test (cut off of 16 and 50, respectively), in dogs that were treated at public veterinary hospitals in the metropolitan region of São Paulo and risk factors were identified. Out of the 1,194 samples 125 (10.5%; 95% CI: 8.8-12.3%) were positive for *T. gondii* and 9 (0.75%, 95% CI: 0.34-1.4%) for *N. caninum*. For *T. gondii*, statistical differences were observed between the proportions of positive dogs and different zones of the municipality (p = 0.025), and age (p = 0.02), higher among older dogs. The keepers were invited to answer an epidemiological questionnaire to analyze risk factors, and 471 (39.4%) agreed to be interviewed, and among their dogs 65 (13.8%) were *T. gondii* seropositive. Age group above 8 years (OR = 3.63; 95% CI: 1.08-12.23) was a risk factor and having a defined breed (OR = 0.49; 95% CI: 0.25-0.96) was a protective factor for *T. gondii* infection. Because of the low number of dogs positive for *N. caninum*, risk factors for this coccidium were not determined.

## Resumo

Cães podem ser infectados por *Toxoplasma gondii* e *Neospora caninum*, os quais funcionam, respectivamente, como hospedeiros intermediários e definitivos. Neste estudo, a soroprevalência contra anticorpos anti-*T. gondii* e *N. caninum* foi determinada pelo teste de imunofluorescência indireta (ponto de corte de 16 e 50, respectivamente), em cães atendidos em hospitais públicos veterinários da região metropolitana de São Paulo e fatores de risco foram identificados. Das 1.194 amostras, 125 (10,5%; IC 95%: 8,8-12,3%) foram positivas para *T. gondii* e 9 (0,75%, IC 95%: 0,34-1,4%) para *N. caninum*. Para *T. gondii* foram observadas diferenças entre as proporções de cães positivos e diferentes zonas do município (p = 0,025) e idade (p = 0,02), prevalência maior entre os mais velhos. Os tutores foram convidados a responder um questionário epidemiológico para análise de fatores de risco, e 471 (39,4%) concordaram em ser entrevistados, destes 65 cães (13,8%) eram soropositivos para *T. gondii*. Faixa etária acima de 8 anos (OR = 3,63; IC 95%: 1,08-12,23) foi fator de risco e raça definida (OR = 0,49; IC 95%: 0,25-0,96) foi fator de proteção para a infecção por *T. gondii*. Devido ao baixo número de positivos para anticorpos anti-*N. caninum*, fatores de risco para este coccídio não foram determinados.

## Introduction

Toxoplasmosis is a cosmopolitan zoonosis caused by the obligate intracellular coccidium *Toxoplasma gondii* (phylum Apicomplexa, family Sarcocystidae). Felines are the only definitive hosts of *T. gondii*, but the disease has complex epidemiology, infecting a wide variety of homeothermic species that behave as intermediate hosts (Dubey, 2016).

In humans, *T. gondii* infections are generally asymptomatic but establish persistent latent infection in tissues and severe clinical disease, even fatal, can be observed, mainly in neonates and immunocompromised individuals (Montoya & Liesenfeld, 2004; Murat et al., 2013). In dogs and cats, *T. gondii* infection is associated with low rates of morbidity and mortality but important clinical consequences may be observed. Toxoplasmosis in dogs is generally linked with immunosuppression and absence of vaccination against canine distemper virus, however neurological and ocular diseases and cutaneous manifestations were already observed in dogs (Dubey & Jones, 2008; Calero-Bernal & Gennari, 2019; Kolören & Dubey, 2020).

The seroprevalence of *T. gondii* antibodies in dogs in Brazil ranges from 3 to 90% (Dubey et al., 2012). In the municipality of São Paulo, the biggest city in this country, the occurrence rate among 118 stray dogs was found to be 35.8% (Dubey et al., 2007). In another study in the same municipality, anti-*T. gondii* antibodies were found in 219 (19.7%) out of 1,110 dogs, among which 610 were stray and 500 were domiciled, with occurrence rates of 31.6% and 5.2%, respectively (Souza et al., 2003), however these studies have more than 10 years and the majority of the samples were from stray dogs that normally have much higher chance to be infected by parasites.

Schares et al. (2005) found viable oocysts in feces of dogs that had ingested cat feces. Furthermore, from examination of fecal samples from 120 dogs, Munoz & Mayer (2016) found that four of them were positive for *T. gondii* DNA, due to some dogs’ habit of ingesting feces. *Toxoplasma gondii* oocysts can be mechanical transmitted to humans by the dogs, by contact with oocysts that may be present in the coat of dogs (Lindsay et al., 1997; Frenkel et al., 2003). Etheredge et al. (2004), in a study with children that have contact with dogs, cats and soil, found that play with dogs was a risk factor to *T. gondii* seropositivity, confirming the importance of dogs as mechanical source of oocysts.

*Neospora caninum* is an obligate intracellular cyst-forming protozoon of the phylum Apicomplexa that causes neosporosis. This coccidium affects domestic and wild animals, with special importance in dogs and cattle (Dubey et al., 2017). The definitive hosts are dogs (McAllister et al., 1998) and some species of wild canids, such as coyotes (Gondim et al., 2004), dingoes (King et al., 2010) and gray wolves (Dubey et al., 2011). It is not considered to be a zoonotic agent, even though antibodies against *N. caninum* have already been found in humans (Lobato et al., 2006; Oshiro et al., 2015; Duarte et al., 2020) and DNA in blood samples from human umbilical cord (Duarte et al., 2020), however the parasite has not yet been detected in tissues.

Numerous studies on occurrences of *N. caninum* antibodies in dogs have been conducted in Brazil and have found rates ranging from 2.6% to 67.6% [reviewed by Cerqueira-Cézar et al. (2017)]. Gennari et al. (2002), in the city of São Paulo, found occurrence rates of 10% in domiciled dogs and 25% in stray dogs.

In 2012, the first public veterinary hospital in Brazil was inaugurated in the municipality of São Paulo. By 2022, there were four hospital units providing free clinical and surgical care for dogs and cats in the metropolitan region of São Paulo (Navarro, 2015).

The state of São Paulo, Brazil, has a population of approximately 13 million dogs and the state capital, São Paulo, around 3,5 million dogs (ABINPET, 2021), and there are no studies using the dog’s population of all the zones of the municipality. The objective of this study was to determine the seroprevalences of *T. gondii* and *N. caninum* and risk factors for these parasites infection among domiciled dogs that are taken to public veterinary hospital services located in different regions of the metropolitan region of São Paulo.

## Materials and Methods

### Sample collection

All samples used in the present study were from dogs attended in Public Hospitals, however the real economic status of the families was not possible to be obtained, however they represent a wide number of animals that live with families that cannot afford to pay a private veterinary service.

Sampling was conducted according to convenience, between August 2021 and July 2022, among dogs that were treated at public veterinary hospitals in the metropolitan region of São Paulo, regardless of clinical suspicion and symptoms. The samples came from four hospitals: three located in the municipality of São Paulo, in the districts of Tatuapé (EZ - east zone), Casa Verde (NZ - north zone) and Jurubatuba (SZ - south zone); and one hospital located in the municipality of Osasco, which borders the west zone (WZ) of the state capital. At the time of obtaining the samples, files containing data about the animal (name, breed, sex, and age) and the owner (name and address) were also created.

The owners of all the study animals were contacted and invited to participate in the study through answering an epidemiological questionnaire, to gain more information than what was available from the clinical files. In the total 1,194 samples were obtained (364 from EZ, 214 from NZ, 250 from SZ and 366 from WZ) and 471 owners accepted to answer the questionnaire (167, 75, 121 and 108, respectively from East, North, South, and West zone).

The distribution between the gender was uniform, with totals of 544 males (45.6%) and 650 females (54.4%). Mixed-breed dogs accounted for the highest percentage of the sampling, with 906 animals (75.9%), while the other 288 (24.1%) were dogs of different breeds. The youngest animal sampled was four months old and the oldest was 20 years old.

### Serum antibody analyses

Serum samples were analyzed by means of the indirect fluorescent antibody test (IFAT) for detection of antibodies against *T. gondii*, as described by Camargo (1974), and against *N. caninum* (Dubey et al., 1988), using cutoffs of 16 and 50, respectively (Valadas et al., 2010). Tachyzoites from the *T. gondii* RH strain, maintained in mice, and the *N. caninum* NC-1 strain, maintained in cell cultures, were used as antigens. Anti-dog IgG conjugate (Sigma®, USA), obtained from rabbits and labeled with fluorescein isothiocyanate, was used at a dilution of 1:600 in phosphate buffer solution (PBS) at pH 7.2, containing 0.01% Evan’s blue. On each slide, positive and negative control serum was added. Reactive *T. gondii* and *N. caninum* serum samples were titrated at base two until the last positive dilution.

### Application of epidemiological questionnaire

After samples had been received, the keepers of the animals that had been examined were contacted by telephone and/or text message, regardless of the results obtained from the samples. Out of the total number of keepers invited to provide additional information, 471 of them agreed to participate in the risk factor analyses and answered a questionnaire containing questions relating to *T. gondii* and *N. caninum* epidemiology. The interview was always done by the same person.

### Statistical analysis

The statistical analysis of this study has two main objectives: first, to estimate the seroprevalences of anti-*T. gondii* and anti-*N. caninum* antibodies and their respective confidence intervals; second, to analyze the association between the presence of anti-*T. gondii* antibodies and potential risk factors.

Confidence intervals for the estimates of the seroprevalences of anti-*T. gondii* and anti-*N. caninum* antibodies were calculated according to zone, age group, sex, breed and in total, using the exact binomial distribution. A comparison of the proportion of positives between the categories of the factors (zone, age group, sex and breed) was performed using the χ^2^ test. In these analyses, data referring to all animals (n = 1194) were used. The significance level adopted in the comparisons was 5%.

To study the association between the presence of anti-*T. gondii* antibodies and risk factors, data from interviews were used (n = 471). The following variables were analyzed: sex, age group (under 1 year old, between 1 and 8 years old or over 8 years old), breed (with or without defined breed), zone (west, south, east or north), access to streets (yes or no), contact with other animal species (no contact or contact with dogs, cats or other animals) and food (commercial, homemade or mixed). An exploratory analysis on the data (univariate analysis) was performed to select the variables for use as part of the logistic regression model, using the χ^2^ test or Fisher's exact test, with a selection criterion of p ≤ 0.20. Statistical analyses were performed using the R language, version 4.2.2 (R Core Team, 2022), and the JASP program, version 0.16.2 (JASP Team, 2022).

## Results

### Toxoplasma gondii

**Figure 1:**
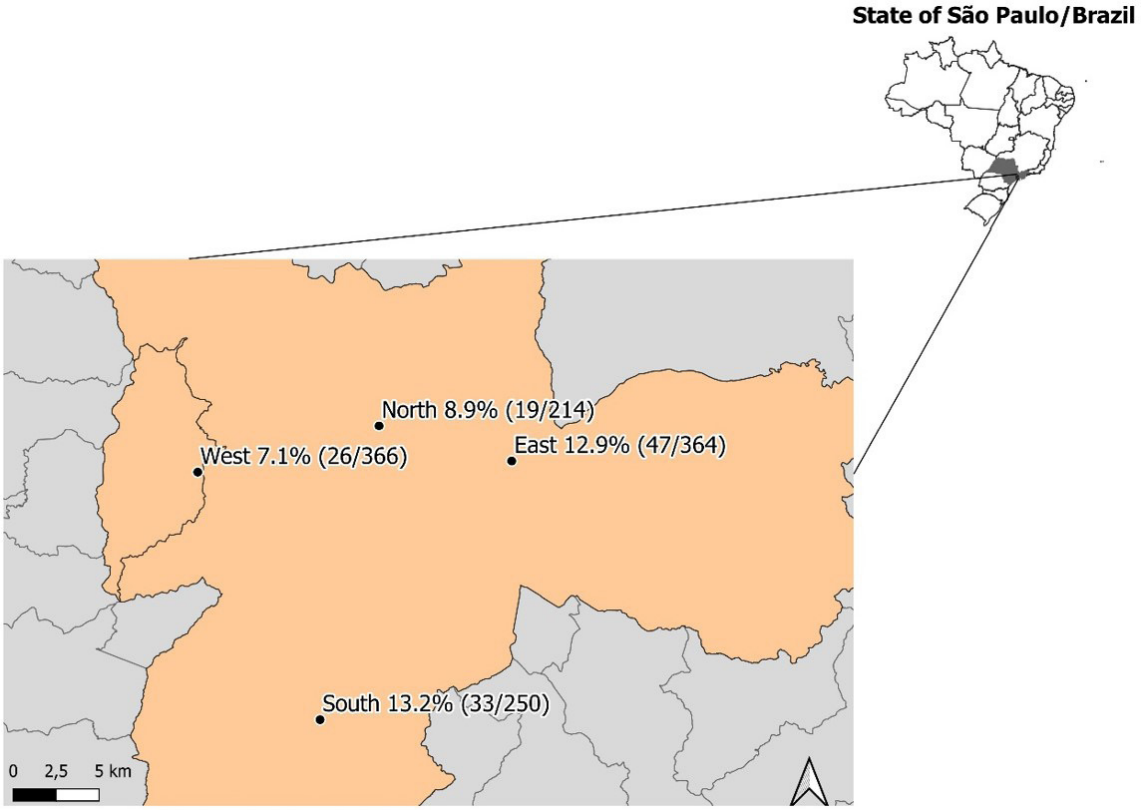
Location of the Public Veterinary Hospital, in the municipality of São Paulo, Brazil, where the dog’s serum samples were obtained, and prevalence values of anti-*Toxoplasma gondii* antibodies.

In the present study, the prevalence of positivity for anti-*T. gondii* antibodies among the dogs sampled was 10.5% (95% CI: 8.8% - 12.3%), i.e., 125 out of the 1,194 animals examined were reactive. The IgG antibody titers were: 32 (n = 51), 64 (n = 26), 128 (n = 25), 256 (n = 10), 512 (n = 4), 1024 (n = 2), 2048 (n = 5) and 4096 (n = 2). The prevalence results according to place of origin of the dogs are shown in in Figure 1 and Table 1.

**Table 1:** Prevalence (95% confidence interval, CI) of *Toxoplasma gondii* antibodies in dogs treated at Public Veterinary Hospitals in São Paulo, Brazil, 2022.

**Variable**	**No. of samples (%)**	**No. of positives**	**Prevalence (95% CI)**
**Zone**			
East	364 (30.5)	47	12.9% (9.6% - 16.8%) ^a^
North	214 (17.9)	19	8.9% (5.4% - 13.5%) ^a,b^
South	250 (20.9)	33	13.2% (9.3% - 18.0%) ^a^
West	366 (30.7)	26	7.1% (4.7% - 10.2%) ^b^
**Age group (years)**			
< 1	144 (12.1)	10	6.9% (3.4% - 12.4%) ^a^
1 to 8	433 (36.2)	36	8.3% (5.9% - 11.3%) ^a^
> 8	617 (51.7)	79	12.8% (10.3% - 15.7%) ^b^
**Sex**			
Male	544 (45.6)	63	11.6% (9.0% - 14.6%) ^a^
Female	650 (54.4)	62	9.5% (7.4% - 12.1%) ^a^
**Defined breed**			
Yes	288 (24.1)	38	13.2% (9.5% - 17.7%) ^a^
No	906 (75.9)	87	9.6% (7.8% - 11.7%) ^a^
**Total**	**1,194 (100.0)**	**125**	**10.5 (8.8% - 12.3%)**

Different letters in the same column – statistically significant.

#### Questionnaire and risk factors

Out of the 1,194 owners, 471 (39.4%) agreed to answer the questionnaire and, among their dogs, 65 (13.8%) were positive for anti-*T. gondii* antibodies. The interview data were used to assess risk factors for *T. gondii* infection. The results from the univariate analysis are presented in Table 2. The selected variables were age group (p = 0.006), breed (p = 0.020), access to the street (p = 0.132) and diet (p = 0.074).

**Table 2:** Results from univariate analysis regarding possible risk factors for the presence of anti-*T. gondii* antibodies in dogs (n = 471) in the metropolitan region of São Paulo, SP, Brazil, 2022.

**Variable and category**	**Negative**	**Positive (%)**	**Total**	**χ^2^**	**p-value**
**Sex**				0.59	0.443
Female	233	34 (12.7)	267		
Male	173	31 (15.2)	204		
**Age group (years)**				10.1	**0.006** *
< 1	49	3 (5.8)	52		
1 to 8	160	17 (9.6)	177		
> 8	197	45 (18.6)	242		
**Defined breed**				5.4	0.020*
No	273	53 (16.2)	326		
Yes	133	12 (8.3)	145		
**Zone**				2.6	0.453
West	89	19 (17.6)	108		
South	104	17 (14.0)	121		
East	145	22 (13.2)	167		
North	68	7 (9.3)	75		
**Contact with animals**				1.8	0.615
No	288	50 (14.8)	338		
Dogs	70	7 (9.1)	77		
Cats	38	6 (13.6)	44		
Others	10	2 (16.7)	12		
**Street access**				2.3	0.132*
Yes	270	37 (17.1)	307		
No	136	28 (12.1)	164		
**Diet**				5.2	0.074*
Commercial	243	48 (16.5)	291		
Mixed	118	14 (10.6)	132		
Homemade	45	3 (6.2)	48		

* Selected for multivariate analysis.

The results from the multiple logistic regression analysis are shown in Table 3. The final logistic regression model for the prevalence of anti-*T. gondii* antibodies indicated that the age group above 8 years (OR = 3.63; 95% CI: 1.08 – 12.23) had a higher chance of being positive for *T. gondii* antibodies than did the other age groups. Furthermore, it indicated that defined-breed animals had a lower chance of being positive for *T. gondii* antibodies than did mixed-breed animals (OR = 0.49; 95% CI: 0.25 – 0.96).

**Table 3:** Results from multiple logistic regression analysis on risk factors for the presence of anti-*Toxoplasma gondii* antibodies in domestic dogs in the metropolitan region of São Paulo, SP, Brazil, 2022.

**Variable**	**Category**	**Odds ratio (OR)**	**95% CI of OR**	**p-value**
**Age group (years)**	< 1*	1.00	–	
	1 to 8	1.77	[0.50-6.33]	0.377
	> 8	3.63	[1.08-12.23]	**0.037**
**Defined breed**	No	1.00	–	
	Yes	0.49	[0.25-0.96]	**0.037**

* Reference category.

### Neospora caninum

Out of the 1,194 serum samples analyzed, 9 (0.75%; 95% CI: 0.34% - 1.4%) were positive for anti-*N. caninum*, with IgG antibody titers of 50 (n = 1), 100 (n = 1), 200 (n = 4), 400 (n = 2) and 800 (n = 1).

All the dogs that were positive for anti-*N. caninum* antibodies were negative for *T. gondii*. Because of the low prevalence observed, no statistical analysis on the findings regarding *N. caninum* was carried out and these results are only presented descriptively (Table 4).

**Table 4:** Prevalence of dogs positive for anti-*N. caninum* antibodies in dogs treated at Public Veterinary Hospitals in São Paulo, Brazil, 2022.

**Variables**	**No. of samples**	**No. of positives**	**Prevalence (%) (95% CI)**
**Zone**			
East	364	5	1.4 (0.4%-3.2%)
North	214	1	0.5 (0.01%-2.6%)
South	250	1	0.4 (0.01%-2.2%)
West	366	2	0.5 (0.07%-1.9%)
**Sex**			
Female	650	3	0.5 (0.1%-1.3%)
Male	544	6	1.1 (0.4%-2.4%)
**Age group (years)**			
<1	144	0	0.0 (0.0%-2.5%)
1 to 8	433	2	0.5 (0.06%-1.7%)
>8	617	7	1.1 (0.5%-2.3%)
**Defined breed**			
Yes	288	3	1.0 (0.2%-3.0)
No	906	6	0.7 (0.2%-1.4%)
**TOTAL**	**1,194**	**9**	**0.75 (0.34%-1.4%)**

## Discussion

In the present study, the prevalence of positivity for anti-*T. gondii* antibodies among the dogs sampled was 10.5% indicating that domestic dogs in the metropolitan region of São Paulo are exposed to infection by this coccidium. In the same city, a study carried out among domestic and stray dogs, found rates of 5.2% (26 out of 500) and 31.6% (193 out of 610), respectively, and it was used the same methodology and cut-off point (Souza et al., 2003). These rates were lower than what was observed in the present study among domiciled dogs.

However, this proportion of positivity was lower than those found by Meireles et al. (2004) and Dubey et al. (2007) among stray dogs in the same city, with rates of 50.5% (101 out of 200) and 35.8% (42 out of 118), respectively. However, it is worth remembering that these comparisons must be made with care, since the diagnostic methods were not similar, and the dogs in the present study were all domiciled.

In a recent review of *T. gondii* in dogs worldwide, Dubey et al. (2020) reported occurrence rates in studies carried out between 2009 and 2020 ranging from 1.9% to 97%; with Brazilian studies values ranging from 6.7% to 88.5%. The authors commented that although several studies have been conducted in Brazil, few of them included more than 300 dogs, however high occurrence rates were observed in the Brazilian studies when compared with results from dogs of other parts of the world, and comparisons between them can be made securely since the same diagnostic test (IFAT) and same cutoff point (16) were used in most of the Brazilian studies.

In the state of São Paulo, seroepidemiological surveys carried out among dogs over the last 20 years have used very different samples, regarding the number of dogs, whether neurological problems were presented or not, where the dogs were living (rural or urban environment) and whether data collection was in connection with a vaccination campaign, among other different characteristics. In these studies, the occurrence rates for anti-*T. gondii* antibodies ranged from 5.1% to 50% (Silva et al., 2010; Aguiar et al., 2012; Langoni et al., 2012, 2013; Paulan et al., 2013; Zanette et al., 2014; Seabra et al., 2015; Olbera et al., 2020; Sevá et al., 2020).

The low prevalence found among domiciled dogs in the present study, even among those that were allowed out into the streets, is an interesting result because it indicates that few dogs have contact with *T. gondii* oocysts in the environment or with cysts in infected meat.

A recent study carried out in the metropolitan region of Rio de Janeiro (Arruda et al., 2021) also evaluated domiciled dogs in different areas of the city. They found a prevalence of 34% (136/400), i.e., three times higher than what was observed in the metropolitan region of São Paulo, also using IFAT (>16) as diagnostic test.

Regarding the zone of São Paulo in which the samples were collected, the analysis on all the sampled animals (n = 1,194) showed that the prevalence in the EZ and SZ were statistically higher, however this information should be analyzed with care once it is not possible to affirm that the dogs always lived in the same region and that they acquire the infection in the region where they live when sampled. Also, the present results need future studies trying to get information about the socio-economic status of the families that use the public veterinary hospitals in the different zones of the city and the habits of the animals. The findings indicate that *T. gondii* is distributed throughout the region studied. This result is like what was found by Arruda et al. (2021) in the metropolitan region of Rio de Janeiro: from analysis on the spatial distribution of the occurrence of parasitized dogs, they observed that positive animals were distributed among all the locations studied.

In the present study, none of the positive dogs showed antibody titers of 16. The most common titer was 32, which was seen in the cases of 51 positive dogs. There were 9 animals with titers of 512. The finding of a greater number of dogs with low antibody titers was also reported from a study on domestic dogs treated at a veterinary hospital in Cuiabá, MT, Brazil (Strital et al., 2016) and from a study on domestic dogs in the city of Rio de Janeiro, RJ, Brazil (Arruda et al., 2021). According to Strital et al. (2016), titers from 16 to 512 can be considered to be contact titers and not situations of acute infection, i.e., they indicate previous contact with the parasite. In the present study, high antibody titers (1024, 2048 and 4096) were found in 9 samples and, given that these were obtained from dogs that were receiving hospital care, it can be assumed that some of them may have been obtained from dogs presenting conditions that lead to immunosuppression, such as ehrlichiosis or distemper, which are quite common in Brazil and which have already been correlated with *T. gondii* infection (Calero-Bernal & Gennari, 2019), however it was not possible to assume that the animals were presenting those infections by the files.

Although neurological conditions have also been correlated with toxoplasmosis (Langoni et al., 2012; Aguiar et al., 2012), none of the *T. gondii* positive dogs in the present study were showing nervous symptoms according to the descriptions found in the veterinary files. However, the information in these files is often superficial, considering that these files do not have the objective of providing all clinical observations.

Multivariate analysis was carried out using data from 471 dogs whose keepers answered the epidemiological questionnaire. The following variables were used: sex, age group, having a defined breed or not, area where the samples were obtained, contact with other animals and diet. Logistic regression pointed to age group (> 8 years old) as a risk factor and having a defined breed as a protective factor for infection by *T. gondii*. Out of the 471 dogs used for these analyses, 65 (13.8%) were seropositive for *T. gondii*, a result very similar to that found from the total sample (10.5%).

As already observed in other Brazilian studies in which domestic dogs were also sampled (Guimarães et al., 2009; Moura et al., 2009; Dantas et al., 2013; Langoni et al., 2013; Benitez et al., 2017; Oliveira et al., 2019), older animals in the present study were found to be more reactive to *T. gondii*. The oldest dogs (> 8 years old) presented the highest occurrence of *T. gondii* infection. This was probably due to the greater chance of exposure to the agent with increasing age.

Regarding the breed of the dogs, having a defined breed was a protective factor against *T. gondii* infection. Brito et al. (2002), Guimarães et al. (2009) and Moura et al. (2009) observed, in studies carried out in Brazil among domestic dogs, that not having a defined breed was a risk factor for infection. They attributed this finding to the likelihood that the owners of these dogs provided better veterinary care for their pets and took better care regarding the food offered to them.

Only 9 (0.75%; 95% CI: 0.34% - 1.4%) of the 1194 serum samples examined were reactive regarding anti-*N. caninum*. Another study carried out on dogs from the same municipality by Gennari et al. (2002), which included both domiciled dogs (n = 500) and stray dogs (n = 600) dogs, found much higher values: respectively 10% and 25%. However, the diagnostic method used was the *Neospora* agglutination test (NAT > 25), which therefore makes it difficult to compare the results.

The values of antibody titers against *N. caninum* ranged from 50 to 800, and 200 was the most frequent titer. Immune Fluorescent Reaction is the test most used to search for antibodies against this coccidium, and the dilution cutoff point of 1:50 is recommended for this. This cutoff point was used in 55 of the 68 studies on the occurrence of *N. caninum* among dogs in Brazil. This test and cutoff point are considered standard for this species [reviewed by Cerqueira-Cézar et al. (2017)].

In Brazil, a series of studies carried out on dogs to search for antibodies against *N. caninum* found higher prevalence than those observed in the present study, ranging from 3.1% to 67.6% (Cerqueira-Cézar et al., 2017). Dogs from urban environments showed lower occurrence rates than those from rural or semi-rural environments. This indicated that urban dogs had fewer opportunities for contact with the infective forms of the parasite, which are usually present in aborted bovine fetuses, fetal membranes, carcasses and prey (Mineo et al., 2004; Fridlund-Plugge et al., 2008; Cunha et al., 2008; Moraes et al., 2008; Valadas et al., 2010; Sousa et al., 2012; Sicupira et al., 2012; Bruhn et al., 2012; Raimundo et al., 2015). According to the information obtained through the epidemiological questionnaire, all the dogs sampled only frequented urban environments.

Due to the low prevalence observed, no statistical analysis on the findings regarding *N. caninum* was carried out. Thus, the results are only presented descriptively. However, despite the low prevalence, positive dogs were found in all regions: both males and females and with and without defined breed. None of the dogs younger than one year old had antibodies against *N. caninum*, and seven of the nine positive dogs were older than eight years of age, thus indicating, in the same way shown earlier regarding *T. gondii*, that the prevalence increased with increasing age of the animals. Other studies carried out in Brazil and in other countries have also observed higher prevalence among older animals (Andreotti et al., 2004; Oliveira et al., 2004; Capelli et al., 2004).

None of the dogs that were seropositive for *N. caninum* were described as having any neurological problems in the records made by the veterinarians who had attended them. Studies on dogs carried out in various parts of the world have shown that although the serum prevalence of antibodies in dogs can be relatively high, clinical disease is relatively rare. Little is known about why some dogs develop clinical symptoms and others do not. This might be due to differences in the virulence of isolates, or due to differences in host susceptibility, or both factors could be involved. Clinical symptoms of neosporosis are usually observed in very young, very old or immunosuppressed dogs (Dubey et al., 2017).

From the results of the present study, it can be concluded that in all zones of the metropolitan region of São Paulo, there are domestic dogs that are positive for IgG anti-*T. gondii* antibodies, with less occurrence in the western region. The risk factors for *T. gondii* infection were age (such that older dogs were more reactive) and mixed breed.

The prevalence of anti-*N. caninum* was less than 1%, but positive dogs were detected in all areas of the municipality.
